# Platelet-Rich Plasma in Alopecia areata: A Case Report With a Mini Review of Literature

**DOI:** 10.7759/cureus.38751

**Published:** 2023-05-09

**Authors:** Lidiya N Todorova, Tsvetana I Abadjieva

**Affiliations:** 1 Dermatology and Venereology, Medical University of Plovdiv, Plovdiv, BGR

**Keywords:** bald spot, hair loss, prp, platelet-rich plasma, alopecia areata

## Abstract

A 46-year-old patient with extensive patchy alopecia areata (AA) treated successfully with platelet-rich plasma (PRP) is presented. The therapy was used in three applications at monthly intervals. The treatment results were analyzed with clinical photography, quantitative assessment of scalp hair, digital trichoscopy, and evaluation of the patient’s quality of life. Results of studies conducted with PRP therapy in alopecia areata are briefly presented. PRP injections in alopecia areata are a relatively effective, safe, low-pain, and minimally invasive treatment method.

## Introduction

Alopecia areata (AA) is a type of non-scarring hair loss affecting most often the scalp. The clinical manifestations of alopecia areata vary from well-defined patches of hair loss (alopecia parcialis) to involvement of the entire scalp (alopecia totalis) or all hairy areas (alopecia universalis) [1]. The course of the disease is unpredictable; spontaneous hair regrowth and subsequent relapse can occur at any time.

The recurrence rate is generally high, and hair loss may persist [2,3]. Recent insights on the etiopathogenesis of AA suggest a genetic predisposition to the disorder and environmental factors, including viral infections, trauma, and psychosocial stress. The loss of the growing hair shafts is linked to the CD4+ and CD8+ T-lymphocytes, which violate the immune privilege of the anagen hair follicle [4]. AA affects patients of both sexes and all ages, with a peak incidence in the second and third decades of life. The condition has approximately 0.1% to 0.2% prevalence in the general population [1].

The case presented in this report is a woman with extensive patchy alopecia areata (AA) treated successfully with PRP. There are published studies, several case reports on the application of PRP in AA, and the results of the studies are briefly presented. However, this treatment is rarely used. Since treatment options in alopecia areata are limited, PRP therapy should be considered.

## Case presentation

A 46-year-old female presented with complaints of patchy hair loss for 17 months. She reported severe stress before the appearance of the bald patch. The patient was treated with hair-growth-promoting shampoos and essential oils with no result. Her condition was stable and did not show any change after the bald area was formed. She used a hairpiece. The patient was in good general health. The bald patch was localized in the right parietal area, 15/8 cm in size, with an irregular oval shape, normal color, mild desquamation, and slight vellus hair regrowth. Hematological and biochemical tests were within normal ranges.

Autologous PRP was applied in three sessions at monthly intervals. For each session, 8-10 ml blood was drawn, collected in a Pure PRP tube, and centrifuged for 10 minutes at 3500 rpm. The resulting 5-6 ml of PRP were injected into the scalp in the sub-follicular plane through multiple intradermal injections. After the third session, increased hair density and scalp coverage was observed, with a 5/4 cm alopecic patch with vellus hairs.

**Figure 1:**
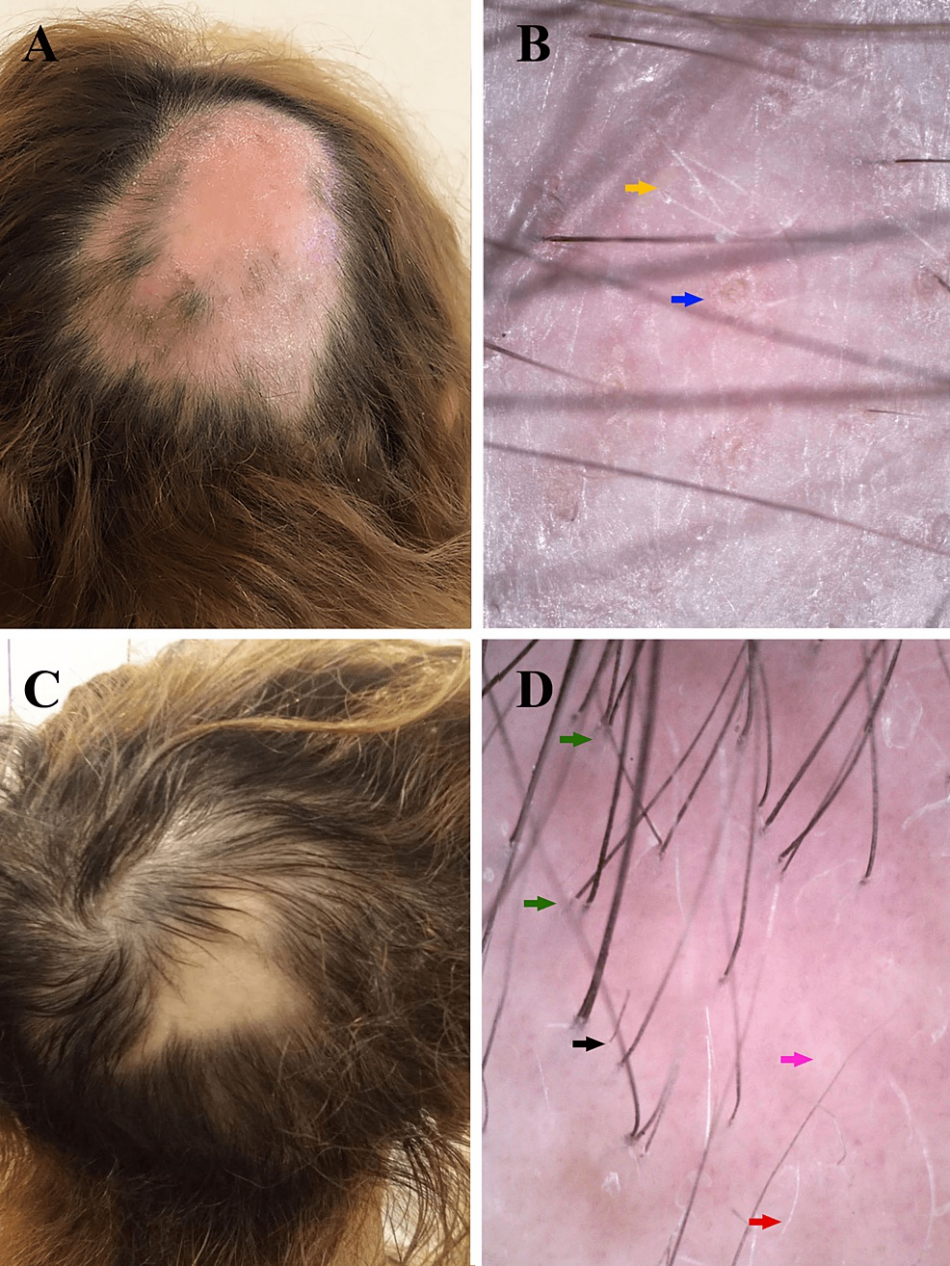
Clinical pictures and trichoscopy images before and after therapy Figure 1. A) Clinical picture before therapy: 15/8 cm sized bald patch in the right parietal area. B) Trichoscopy findings before therapy: yellow dots (blue arrow), short vellus hairs (yellow arrow). C) Clinical picture a month after the last treatment: 5/4 cm sized bald patch in the right parietal area. D) Trichoscopy findings a month after the last treatment: upright regrowing hairs (green arrows), vellus hairs (red arrow), minimal broken hairs (black arrow), and solitary yellow dots (pink arrow).

The treatment results were analyzed with clinical photography, assessment of scalp hair loss, digital trichoscopy, and quality of life evaluation at two-time points: at the beginning of therapy (T1) and a month after the last treatment (T2). The quantitative assessment of scalp hair loss was done via the Severity of Alopecia Tool (SALT) Score [5]. Dermatology Life Quality Index (DLQI) was used to evaluate the patient’s quality of life [6]. The SALT score was 18% at T1, the trichoscopy revealed predominantly yellow dots and short vellus hairs, and the DLQI score was 20. At T2, the SALT score decreased to 5%, and the DLQI score to 10. Trichoscopy findings at T2: a significant number of upright regrowing hairs, longer, thicker, and pigmented vellus hairs, red dots, minimal broken hairs, and solitary yellow dots. Trichoscopy was performed with Dino-Lite Edge Digital Microscope AM7915MZT(R7), magnification x70 (Figure 1).

## Discussion

Objective assessment of treatment efficacy is difficult due to the high rate of spontaneous remission. Visible hair growth was established after the first PRP session. The effect of the therapy was sustained, and there was no hair loss, and full hair regrowth was achieved at follow-up over the next two years. The results clearly showed substantial hair recovery, evidenced by SALT score, photography, and trichoscopy. Trichoscopy may serve as therapeutic monitoring of the disorder [7]. After the PRP therapy, the DLQI showed a 50% decrease, indicating the improved patient’s quality of life.

Therapeutic options for AA have limited and uncertain success. Traditional treatment options include corticosteroids, immunomodulators, minoxidil, and contact immunotherapy. Current insights into the pathogenesis of AA have led to the introduction of Janus kinase (JAK) inhibitors, regarded as a promising treatment option for AA. Several clinical studies investigated the efficacy and safety of JAK kinase inhibitors for AA patients. The majority responded with hair regrowth, although they may experience hair loss after cessation of therapy. Other modern treatments for AA include antihistamines, PRP, and microneedling [4].

PRP is a platelet-concentrated autologous plasma used to stimulate hair growth and follicle regeneration. Platelets secrete more than 20 important growth factors and cytokines, including platelet-derived growth factor (PGDF), vascular endothelial growth factor (VEGF), transforming growth factor (TGF), fibroblast growth factor (FGF), connective tissue growth factor, epidermal growth factor (EGF), and Insulin-like Growth Factor I (IGF-1). They stimulate hair follicle proliferation, induce angiogenesis, and develop adnexal structures. In addition, PRP has a potent anti-inflammatory effect and can suppress cytokine release and limit local inflammation. AA is characterized by an inflammatory infiltrate and secretion of various inflammatory cytokines. Therefore, the anti-inflammatory properties of PRP may greatly benefit the condition [7-9]. PRP was also found to significantly increase hair regrowth and decrease hair dystrophy compared with triamcinolone acetonide or placebo [8]. Other authors have established that PRP is effective in mild cases of AA [10]. PRP was reported to be more effective in treating AA than topical minoxidil 5% [11].

A comparative study established the efficacy of PRP comparable with intralesional triamcinolone for the treatment of AA [9]. Of 20 patients with chronic AA treated with PRP in six sessions at 4 week-intervals and followed for one year, one patient had a relapse and minimal hair growth [12]. In addition, limited effectiveness of PRP treatment in chronic severe AA has been reported. The study found that none of the cases at a one-year follow-up had full hair regrowth, and none achieved noticeable cosmetic results. This suggests that the treatment with PRP is beneficial in a few cases with mild forms of AA and not in severe forms of AA. PRP therapy fails to provide persistent results and inhibit new relapses. Another disadvantage of the treatment is the significant discomfort and pain caused by the injection [13]. PRP application is an intervention with minimal adverse effects: transient pain during injection, temporary erythema and edema, mild headache, and desquamation. The most frequently reported reason for therapy discontinuation, however, is the pain caused during the injection of PRP [7,14]. Contraindications to PRP include coagulation disorders, platelet dysfunction, anticoagulant therapy, thrombocytopenia, hemodynamic instability, local infections at the site of blood harvest or PRP injections, hepatitis, and patients who are prone to keloid formation [14].

## Conclusions

In conclusion, PRP injections in AA are a relatively effective, safe, low-pain, and minimally invasive treatment method. The case presentation is an example of a PRP therapy with a beneficial impact in a chronic severe case of AA. Hair growth stimulation improved the patient’s quality of life. However, this treatment is rarely used. Since treatment options in alopecia areata are limited, PRP therapy should be considered. Larger studies are needed to establish the efficacy and safety of PRP treatment in AA.
